# `This is our country and somehow, we have to make it work’: a sequential explanatory mixed-methods study of the enablers of non-migration and return migration in a cohort of Nigerian medical doctors and dentists

**DOI:** 10.1080/16549716.2026.2623345

**Published:** 2026-03-20

**Authors:** Paul Ikhurionan, Patience Toyin-Thomas, Efetobo V. Orikpete, Philippa Odika, Oti N. Aria, Avwebo O. Ukueku, Yasangra R. Adeniji, Chinelo Iwegim, Uwaila Otakhoigbogie, Itua C. G. Akhirevbulu, Sunday C. Madubueze, Ekhosuehi T. Agho, Chukwunwike W. Ozegbe, Josephine Atat, Oluchi Omogbai, Ekpereka S. Nawfal, Uyoyo Odogu, Oladapo Oladeinde, Efe E. Omoyibo, Ukachi C. Nnawuihe, Oghenebrume Wariri

**Affiliations:** a Institute of Child Health, University of Benin, Benin, Nigeria; b The Department of Pediatrics, Geisel School of Medicine, Dartmouth College, Hanover, NH, USA; c The Dartmouth Institute for Health Policy and Clinical Practice, New Hampshire, Lebanon; d Department of Oral Pathology, Oral Medicine and Oral Radiology, Faculty of Dentistry University of Port Harcourt, Port Harcourt, Nigeria; e Independent Researcher, Sugar Land, TX, USA; f Burns, Plastic and Reconstructive Surgery Unit, Department of Surgery, Rivers State University Teaching Hospital, Port Harcourt, Nigeria; g Department of Obstetrics and Gynaecology, University of Port Harcourt Teaching Hospital, Port Harcourt, Nigeria; h Department of Paediatrics, Federal Teaching Hospital, Gombe, Nigeria; i Medical Microbiology Department, University of Toronto, Toronto, Canada; j Department of Oral Pathology and Oral Medicine, Faculty of Dentistry, College of Medicine, Ituku/Ozalla Campus, University of Nigeria, Enugu, Nigeria; k Orthopaedics and Traumatology Division, Department of Surgery, Edo Specialist Hospital, Benin, Nigeria; l Department of Family Medicine, Federal University Teaching Hospital, (FUTH), Lafia, Nigeria; m Dental and Maxillofacial Surgery Department, National Hospital Abuja, Abuja, Nigeria; n Department, Obstetrics and Gynaecology, Federal University Teaching Hospital, Lafia, Nasarawa State, Nigeria; o Department of Restorative Dentistry, University of Benin Teaching Hospital, Benin, Nigeria; p Independent Researcher, Abbotsford, British Columbia, Canada; q Department of Epidemiology, Florida International University, Miami, FL, USA; r Mike Petryk School of Dentistry, University of Albert, Edmonton, Alberta, Canada; s Department of Family Medicine, Medicine Hat Regional Hospital, Medicine Hat, Alberta, Canada; t Department of Paediatrics, Federal Medical Centre, Asaba, Nigeria; u Department of Public Health, Intercountry Centre for Oral Health for Africa, Jos, Nigeria; v Department of Infectious Disease Epidemiology, London School of Hygiene and Tropical Medicine, London, UK; w Vaccines and Immunity Theme, Medical Research Council Unit the Gambia, London School of Hygiene and Tropical Medicine, Fajara, The Gambia

**Keywords:** Emigration, intention to migrate, return migration, doctors and dentists, mixed-methods

## Abstract

**Background:**

While many studies have explored the drivers of health-worker emigration, there is limited understanding of the factors that potentially encourage them to remain or return after migration.

**Objectives:**

We explored three interrelated questions: what factors encouraged some members of the study population to remain in Nigeria?; what circumstances might encourage those intending to migrate to reconsider their plans?; and what conditions could encourage those who have already emigrated to consider returning?

**Methods:**

We conducted a sequential explanatory mixed-methods study among a cohort of Nigerian-trained doctors and dentists. In the quantitative phase, 274 cohort members completed a structured survey assessing drivers of migration. In the qualitative phase, 50 participants across three migration status groups (emigrated, intending to migrate, and not intending to migrate) were interviewed. Thematic analysis was conducted.

**Results:**

Overall, 49.3% (135/274) of the cohort had already migrated within 15 years of qualifying, while 63.6% (82/139) of those still in Nigeria expressed an intention to migrate. Qualitative findings reinforced the quantitative results, highlighting shared potential enablers of staying (among those intending to migrate) or returning (among already migrated), including improved security, economic stability, better remuneration, stronger healthcare infrastructure, and enhanced training opportunities. Most of those who had already migrated expressed a willingness to return, though often as a long-term plan. Those with no intention to migrate cited a sense of duty and patriotism, family responsibilities, thriving businesses, and professional or age-related factors as reasons for staying back.

**Conclusion:**

This study offers actionable insights to inform policies on health-worker migration.

## Paper Context

- **Main findings:** The main drivers of emigration among doctors who have migrated or intend to do so, are largely macro-level factors such as insecurity, concerns about children’s future, poor remuneration, and high cost of living. On the other hand, micro-level factors such as patriotism, and family responsibilities are the main reasons for non-migration among those who have chosen not to emigrate from the country.
- **Added knowledge:** This mixed-methods study examine the deeper motivations behind non-migration of doctors, the triggers for considering migration, and the potential enablers of return migration, providing a comprehensive comparison of perspectives across three distinct groups of doctors.
- **Global health impact for policy and action:** The results of this study offer timely and policy-relevant evidence to inform national and regional strategies to support the return migration and improve non-migration of skilled health professionals.

## Background

The emigration of highly skilled health workers, particularly doctors, dentists, and nurses, who form the backbone of health systems in low- and middle-income countries (LMICs) poses a growing threat to health equity and service delivery. This ‘brain drain’ severely depletes the health workforce, placing immense strain on already fragile and overstretched health systems [1–3]. The loss of trained professionals not only weakens healthcare infrastructure but also exacerbates existing health disparities, leaving vulnerable populations with limited access to essential services. A 2023 World Health Organization (WHO) report found that 55 of its 194 member states fall below the global median of 49 healthcare workers per 10,000 population, with the most acute shortages occurring in Africa, where 37 out of 54 countries are affected. Nigeria is among the hardest hit, facing a significant health workforce crisis [4].

Nigeria’s health workforce density was an estimated 20 healthcare workers per 10,000 population in 2022, well below the WHO’s recommended threshold [5]. Compounding this shortage, a 2022 report documented that between 2021 and 2022, 13,609 health workers migrated from Nigeria to the United Kingdom [6]. Further evidence from a 2024 study revealed that nearly half of a graduating cohort of Nigerian doctors and dentists had emigrated within 15 years of qualification, underscoring the systemic and enduring nature of the problem [7]. The decade preceding 2024 was marked by overlapping political and economic pressures, including currency volatility, rising inflation, and pervasive insecurity. Between 2015 and 2022, real GDP per capita declined as fiscal deficits widened under weakened oil revenues [8]. Inflation frequently exceeded 20%, severely eroding household purchasing power [9]. Nigeria also ranks among the most insecure countries in Africa; the Armed Conflict Location & Event Data Project (ACLED) recorded approximately 3700 incidents of political violence and 3900 civilian fatalities in 2022 [10].

While previous studies have identified a range of contextual drivers of healthcare worker emigration, these are typically framed as push factors, such as poor working conditions, insecurity, and low remuneration, and pull factors such as better opportunities, career advancement, and safer environments [11]. However, there remains a limited understanding of what compels some healthcare workers to stay, reconsider their plans to emigrate, or potentially return after migrating. Addressing the persistent emigration of Nigerian doctors and dentists requires a more nuanced understanding of these decision-making processes. Although some research has documented rates of emigration and intention to migrate [12–14], and a few studies have proposed strategies to reduce migration [15], most of the research has focused predominantly on the subpopulations of health workers who have already migrated or are actively intending to migrate. Little is known about the motivations of those who remain, or the conditions under which emigrated professionals might consider returning. In addition, the contextual factors that influence these decisions, and how they might inform policy, are still not well understood. This gap in the literature limits the development of holistic and evidence-informed interventions to address the ongoing health workforce crisis.

This explanatory mixed-methods study, grounded in a pragmatist epistemological stance, addresses the identified evidence gaps by focusing on a well-characterized cohort of Nigerian-trained medical doctors and dentists. The pragmatist perspective recognizes that complex social phenomena, such as migration decisions, are best understood through the complementary strengths of quantitative measurement and qualitative interpretation. Accordingly, we investigate three interrelated questions: what factors have encouraged some members of this cohort to remain in Nigeria; what circumstances might encourage those intending to migrate to reconsider their plans; and what conditions could encourage those who have already emigrated to consider returning. The quantitative component examined the proportions of the cohort who had already migrated, intended to migrate, or had chosen to stay back in Nigeria, along with the factors influencing these decisions. Building on these findings, the qualitative component sought to understand these findings by examining the deeper motivations behind staying, the potential enablers for reconsidering migration, and the potential enablers of return migration. By integrating both strands of data, the study provides a comprehensive comparison of perspectives across these three groups.

## Methods

### Study design

We utilized a sequential explanatory mixed-methods design for this study [16]. In the first phase, we collected quantitative survey data from a cohort of Nigerian medical doctors and dentists who graduated from the University of Benin medical school 15 years before commencement of the study. We studied this cohort because it represents a well-defined and traceable group of doctors and dentists who trained together for at least 6 years and have accumulated over 15 years of post-qualification experience. Members originate from and currently work across diverse regions of Nigeria and several countries abroad, offering broad contextual insights into medical migration. In the absence of a national registry of medical graduates, this cohort provided a uniquely accessible and well-characterized population for examining long-term migration patterns among Nigerian-trained medical doctors and dentists.

### Study population and data

#### Quantitative data

Detailed information about the study population, eligibility criteria and the quantitative data collection have been described in a previous publication [7]. In brief, the study population consisted of all 383 medical doctors and dentists who graduated from the University of Benin Medical School in 2008. Four members of the graduating cohort were deceased, leaving 379 eligible participants. Of the 379 eligible cohort members, 298 agreed to participate in the study and received the quantitative questionnaire, with 274 respondents completing and returning the questionnaire.

We used a structured, pre-tested, self-administered questionnaire developed specifically for the study. The questionnaire was developed based on findings from a systematic review of healthcare worker migration, which synthesized studies conducted in LMICs between 1970 and 2022 [11]. Quantitative data collection was conducted using *KoboToolbox®* [17], a secure data collection and management platform. The quantitative questionnaire included sections on demographic characteristics, career trajectory, migration status, and factors influencing migration. The findings from the quantitative survey guided the second phase of the study, where we collected qualitative data to help explain the ‘why’ behind the ‘what’ found in the quantitative phase.

In this study, ‘*intention to migrate*’ was operationally defined as the self-reported expression of an individual’s plan to leave Nigeria to practise or reside abroad among respondents who had not yet migrated. This reflected an expressed intent or interest in migration, irrespective of whether any concrete plans or formal steps (such as applications or job offers) had been initiated. Conversely, ‘*no intention to migrate*’ referred to respondents who explicitly expressed an unwillingness or no immediate plans to leave Nigeria despite currently residing and practicing there. Finally, *‘migrated’* referred to respondents who reported having already left Nigeria to live or work abroad, regardless of the duration of stay or destination country, but excluding those who travelled temporarily for short-term purposes, such as conferences, training, or workshops.

Quantitative data collection was conducted over a six-week period, from 3 March to 20 April 2024. The quantitative questionnaire included an item asking participants whether they would be willing to participate in a follow-up qualitative interview. Of the 274 respondents who completed the questionnaire, 141 indicated interest in participating, while 133 declined.

#### Qualitative data

For the qualitative component of this study, we categorized cohort members into three groups to answer the research questions, based on their migration status at the time of data collection: (1) those who had emigrated from Nigeria; (2) those who were still in Nigeria but intended to migrate; and (3) those residing in Nigeria with no intention to migrate. Using purposive sampling, we recruited participants from each group among individuals who had previously indicated willingness to participate in follow-up interviews in the quantitative survey.

To capture diverse perspectives and lived experiences, we purposively selected 50 participants based on the number needed to achieve saturation, ensuring variation in biological sex, geographic location of residence, and postgraduate clinical training status (i.e. enrolled or completed) (see Table 1 for participant characteristics). Data were collected through one-on-one key informant interviews (KIIs) using a semi-structured interview guide. The guide was developed to explore migration decisions, underlying motivations, and participants’ views on policy solutions to reduce emigration and promote return migration. All the 50 interviews were conducted in English Language over Zoom, between 8 August and 6 October 2024, spanning a period of 8 weeks. Each session lasted approximately 45 min and was scheduled at a time convenient to the participants. Interviews were audio-recorded with informed consent and transcribed verbatim by members of the research team. To ensure transcription accuracy, the interviewer reviewed and validated all transcripts.

**Table 1.** Summary information of the 50 participants included in the key-informant interviews, according to their selection variables.

Selection variables	Emigrated (*N* = 20)	Not migrated from Nigeria (*N* = 30)	
		Intention to Migrate (*N* = 16)	No intention to migrate (*N* = 14)
Sex, ***n*** Male Female	10 10	11 5	8 6
Location, ***n*** ***Overseas*** United Kingdom United States Canada South Africa United Arab Emirates ***Nigeria*** North-west North-east North-central South-west South-east South-south	5 5 7 2 1 NA NA NA NA NA NA	NA NA NA NA NA 0 1 3 2 1 9	NA NA NA NA NA 0 1 1 2 0 8
**Residency status** Completed Residency In training Discontinued training No residency	6 5 4 5	6 5 1 4	4 1 0 9

NA = not applicable.

### Data management and analysis

#### Quantitative data analyses

Quantitative data were analysed descriptively to summarise participant characteristics and survey responses. Frequencies, percentages, and mean scores were calculated to describe demographic variables and migration-related factors. We categorized the quantitative drivers of migration as either push or pull factors. Push factors are conditions that compel individuals to leave their home country (e.g. Nigeria), while pull factors are those that attract them to destination countries [18]. These drivers were further categorized as macro, meso, and micro-level factors, following the conceptual framework developed by Young et al. [19] Macro-level factors reflect global and national influences; meso-level factors relate to professional considerations; and micro-level factors involve personal circumstances shaping migration decisions. Group comparisons were conducted using *t*-tests for continuous variables and chi-square (*χ*^2^) tests for categorical variables. A *p*-value of less than 0.05 was considered statistically significant. All descriptive analyses were performed using Stata version 18 (StataCorp LLC) and all figures were made using the R software (R Foundation for Statistical Computing, 2023) [20,21].

#### Qualitative data analyses

Qualitative data from the KIIs were analyzed thematically using the approach outlined by Braun and Clarke [22]. Transcripts were imported into Delve®, an online qualitative analysis platform [23]. We applied a hybrid coding approach that combined inductive and deductive techniques. The inductive approach enabled the emergence of themes directly from participants’ narratives, while the deductive approach was guided by pre-identified codes derived from the quantitative findings and existing literature [24].

To ensure analytical rigor, multiple authors independently read and re-read the transcripts to become familiar with the content and identify recurring ideas. Initial codes were developed and used to construct candidate themes and sub-themes. The coding framework was iteratively refined through ongoing comparison with the data, assessing internal consistency and relationships among themes. This process continued until thematic saturation was achieved, that is, when no new codes, patterns, or insights emerged from additional interviews, and the existing themes were sufficiently rich and well defined to capture the depth and diversity of participants’ perspectives. Final themes were reviewed and agreed upon collectively by the co-authors. In cases where coding discrepancies arose, a different member of the research team who was not involved in the initial coding was consulted to resolve disagreements and ensure consistency in interpretation.

### Ethics

Ethical approval for this study was obtained from the Research Ethics Committee of the College of Medical Sciences, University of Benin, Edo State, Nigeria (Reference: CMS/REC/2023/457; Date: 23 October 2023). All participants provided written informed consent electronically prior to participation in the quantitative survey. For the qualitative component, additional consent was obtained before each interview. To maintain confidentiality, participants were assigned unique study identifiers, and no personally identifiable information was linked to the data. All data were stored securely in password-protected electronic files accessible only to authorized members of the research team.

## Results

### Characteristics and migration pattern of the cohort

Of the eligible cohort, 72.3% (274/379) of participants completed the quantitative questionnaire. The response rate was 70.1% (225/321) among medical doctors and 84.5% (49/58) among dentists. Overall, 49.3% (135/274) of respondents had already migrated from Nigeria at the time of the study, while 50.7% (139/274) remained in the country (Table 2). Among those who had not migrated, 63.6% (82/139) expressed an intention to migrate, whereas 36.4% (47/139) had no plans to leave Nigeria.

**Table 2.** Characteristics of 2008 cohort of graduates from the University of Benin medical School 15 years post-graduation (i.e. 2008–2024) by migration status.

Variable		Not migrated*			Total not migrated *n* = 139 (50.7%)	Total emigrated *n* = 135 (49.3%)	Overall (*n* = 274)	*P*-value
		No intention n = 47 (36.4%)	Intention to migrate n = 82 (63.6%)	*p*-value				
**Age**								
Median age (years)		43	42		42	41	42	
Mean age (years)		43.4	42.8	*0.234*	43.0	41.8	42.4	***0.0003***
Age range (years)		40–54	39–51		39–54	36–60	(36–60)	
Sex, ***n*** (%)								
Female		16 (34)	23 (28)		45 (32.4)	55 (40.7)	100 (36.5)	
Male		31 (66)	59 (72)	*0.476*	94 (67.6)	80 (59.3)	174 (63.5)	*0.150*
Marital **s**tatus, ***n*** (%)								
Married		40 (85.1)	76 (92.7)		125 (90.6)	121 (89.6)	246 (89.8)	
Not married**		7 (14.9)	6 (7.3)	*0.172*	13 (9.4)	14 (10.4)	25 (9.1)	*0.910*
**Timing of marriage**								
Median year married		2012	2012		2012	2012	2012	
Range (year married)		1993–2021	1998–2021		1993–2021	1996 –2021	1993–2021	
**Dependents**								
Nuclear family: dependents (median)		4.5	5		5	5	5	
Nuclear family: dependents (range)		0–10	1–8		0–10	0–9	0–10	
Extended family: dependents (median)		2	3		3	2	2	
Extended family: dependents (range)		0–16	0–15		0–16	0–21	0–21	
Commenced postgraduate training, ***n*** (%)								
Yes		35 (74.5)	70 (85.4)		114 (82.0)	115 (85.2)	229 (83.6)	
No		12 (25.5)	12 (14.6)	*0.126*	25 (18)	20 (14.8)	45 (16.4)	*0.479*
**Type of postgraduate training**								
***Commenced residency***		25 (71.4)	61 (87.1)	***0.049***	95 (83.3)	101 (87.8)	196 (85.6)	*0.333*
	Completed	19 (76.0)	30 (49.2)	***0.023***	53 (55.8)	63 (62.4)	116 (59.2)	*0.348*
	Currently enrolled	6 (24.0)	28 (45.9)	*0.059*	38 (40.0)	13 (12.9)	51 (26.0)	***0.0001***
	Discontinued	0 (0)	3 (4.9)	*0.259*	4 (4.21)	25 (24.8)	29 (14.8)	***0.0001***
***Commenced Master’s***		13 (37.1)	21 (30.0)	*0.461*	39 (34.2)	35 (30.4)	74 (32.3)	*0.541*
	Completed	8 (61.5)	15 (71.4)	*0.549*	26 (66.7)	31(88.6)	57 (77.0)	***0.025***
	Currently enrolled	5 (38.5)	4 (19.0)	*0.212*	10 (25.6)	4 (11.4)	14 (18.9)	*0.119*
	Discontinued	0 (0)	2 (9.5)	*0.251*	3 (7.7)	0 (0)	3 (4.1)	*0.094*
***Commenced PhD***		3 (8.6)	5 (7.1)	*0.795*	9 (7.9)	8 (7.0%)	17 (7.4)	*0.787*
	Completed	0 (0)	2 (40.0)	*0.206*	3 (33.3)	3 (37.5)	6 (35.3)	*0.858*
	Currently enrolled	3 (100.0)	3 (60.0)	*0.206*	6 (66.7)	4 (50.0)	10 (58.8)	*0.486*
	Discontinued	0 (0)	0 (0)	–	0	1 (2.5)	1 (5.88)	*0.274*
Other training***		4 (11.4)	3 (4.3)	*0.167*	8 (7.0)	8 (7.0%)	17 (7.4)	*0.815*
**Clinical Practice? *n* (%)**								
No		6 (12.8)	8(9.8)		14 (10.1)	30 (22.2)	44 (16.1)	
Yes		41 (87.2)	74 (90.2)	*0.597*	125 (89.9)	105 (77.8)	230 (83.9)	***0.006***
**Percent clinical practice:**								
≤25% clinical practice		2 (4.9)	1 (1.3)		3 (2.4)	2 (1.9)	5 (2.2)	
50% clinical practice		0 (0)	11 (14.9)		11 (8.8)	6 (5.7)	17 (7.4)	
75% clinical practice		14 (34.2)	23 (31.1)		41 (32.8)	12 (11.4)	53 (23.0)	
100% clinical practice		25 (61%)	39 (52.7)		70 (56.0)	85 (81.0)	155(67.4)	

*Ten respondents who have not migrated did not respond to the question on intention to migrate. **Not Married includes people who were single, separated, divorced, or widowed. ***Other postgraduate training includes diploma courses or specialized clinical courses.

The mean age of the study population was 42.4 ± 1.8 years. However, those who had not migrated were older than those who had already migrated (43.0 ± years vs. 41.8 ± 1.7 years, *p* = 0.0003). Among those who had not migrated, a significantly lower proportion of individuals with no intention to migrate had started residency training compared to those intending to migrate (53.2% [25/47] vs. 74.3% [61/82], *p* = 0.049). Furthermore, a significantly higher proportion of those who had already migrated had discontinued their residency training compared to those who had not migrated (24.8% [25/101] vs. 4.2% [4/95], *p* < 0.001). A detailed comparison of the characteristics by terminal degree of the cohort, i.e. medical doctors (i.e. MBBS) vs. dentists (i.e. BDS) is provided in Supplementary Table S1 of the supplementary appendix.

### Quantitative drivers of intention to migrate, non-migration, and return migration

**Figure 1.**
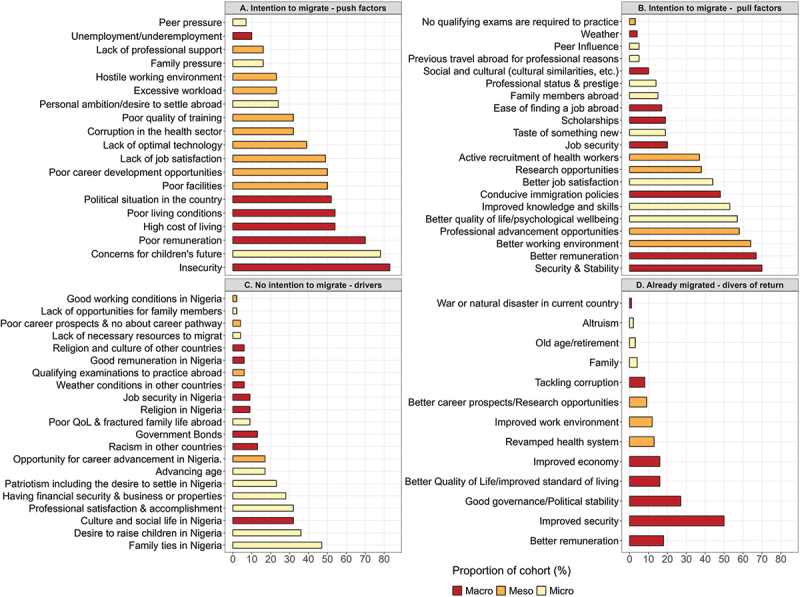
Quantitative factors influencing (A) migration intentions [i.e. push factors], (B) migration intentions [i.e. pull factors], (C) non-migration, and (D) potential return migration among the cohort, categorized by macro-, meso-, and micro-level factors.

Among participants who had not migrated but intended to do so, the most commonly reported push factor was insecurity of lives and property (83%), followed by concerns about their children’s future (78%), poor remuneration (70%), high cost of living (54%), and poor living conditions (54%) (Figure 1(a)). Among these top five push factors, four (80%) were macro-level factors. Similarly, the top three factors attracting individuals with the intention to migrate abroad (i.e. pull factors) were better security (70%), higher remuneration (67%), and an improved working environment (64%) (Figure 1(b)).

QoL = Quality of Life. Proportions are based on the number of individuals in each category, with multiple responses possible.

Among participants who had not migrated and had no intention of doing so, key reasons for staying back in Nigeria included family ties (47%), the desire to raise children in Nigeria (36%), social life in Nigeria (32%), professional satisfaction and accomplishments (32%), and financial security, including business or property ownership (28%). Notably, four of the top five (80%) reasons for non-migration were micro-level factors (Figure 1(c)).

Among participants who had already migrated, the top five potential drivers of return migration were all macro-level factors (Figure 1(d)). These included improved security of lives and property (50%), good governance and political stability (27%), better remuneration (18%), economic improvements (16%), and enhanced living standards (16%).

### Qualitative findings: potential enablers of return migration, reconsideration of migration intention and motivation for staying back in Nigeria

The KIIs explored three interrelated dimensions of migration decision-making: (i) the underlying motivations for staying back in Nigeria among those with no intention to migrate, (ii) the potential enablers of reconsidering migration intentions among those still planning to migrate, and (iii) the potential enablers of return migration among those who had already migrated. Table 3 presents the major themes identified within each group, along with representative quotes.

**Table 3.** Themes and supporting quotes from qualitative key informant interviews showing enablers of return migration, reconsidering migration intentions and motivation for staying back in Nigeria.

Group	Themes	Exemplar quotes
Already migrated (enablers of return migration)	Improvement in security	‘..*the security in Nigeria needs to be improved. I’ve heard of our colleagues being killed, colleagues being kidnapped. How can doctors be afraid of their own security? (p12, Male, USA)’* *‘Well, many people have spoken about security. Iin my own family, we’ve had issues of kidnap. Relatives who were kidnapped, ransom paid and bodies were not even seen … . It’s that sad of a thing. (p27, Female, UK)’*
	Improved economy and better remuneration	*‘the exchange rates of the Naira to the pounds is so bad that no matter how much you’re paid … ,it’s almost as though you’ve got nothing. So, the economic situation needs to be upgraded. (p27, Female, UK)’* *‘one of the things I think that can help with [return] is better renumeration … It’s a very sad thing that doctors have to migrate just to get better pay, when we have a country that needs us. (p28, Female, Canada)’*
	Healthcare system development	*‘There were times when you couldn’t even get simple thing as oxygen saturation monitor … There’s a difference between you knowing what to do and having what you need to do your work. (p08, Male, Canada)’* *‘ … back home, people are overworked because most of our colleagues have left. So, going back presently you are likely going to do the work of three or four people for your basic salary. (p27, Female, UK)’*
	Social infrastructural development	‘*Give me electricity, give me water, give me good roads, … but when I have to struggle for the basics, and struggle for the big things, it becomes too much of a burden. (p16, Female, Canada)’* *‘ … infrastructure, including water and electricity … I feel like if those were improved on, then it will make the idea [of return migration] a bit more bearable and comfortable. (p22, Female, Canada)’*
	Community and family ties	*‘if there’s anything that would want to make me move back, it’s family. My family, my parents are still in Nigeria. I have siblings in Nigeria. (p7, Male, USA)’* *‘I miss being with my siblings, too … we’re heavily reliant on social media to communicate. There are times a good hug from a family member makes all the difference. (p15, Female, UK)’*
Intending to migrate (enablers of reconsidering migration intentions)	Improvement in security	‘*You hear every day of doctors being kidnapped … I know it’s not just doctors being kidnapped and it affects everyone but something needs to be done to reduce this in the country (p09, Female)’* *‘Topping the list is improving the security … There is tension, stories of kidnapping here and there … and it’s one of those things that would make me to consider migrating out of Nigeria. (p33, Female)’*
	Improved economy and better remuneration	*‘Then the other factor is the inflation rates eating into what you think you are getting paid. At the end of the day, what I’m making is not sufficient to run my home. (p21, Male)’* *‘Why not better pay. When I was a house officer, I earned approximately $1,000 and that was in 2009. Now I’m a principal medical officer, and what I earn is not even up to $700 when converted. (p09, Female)’*
	Enhanced healthcare infrastructure	*We do not have the state-of-the-art facilities that can enhance and boost your career development in this country, especially where I’m working … and that has to change. (p33, Female)”* *The primary health care centres are not even functional, the ones that are functional are not even up to standard compared to other countries … something has to be done. (p09, Female)”*
	Improved training and professional development	*‘I am thinking of going to settings where there will be advancements in my skill. You know, the skills that I couldn’t get in Nigeria, I will be able to acquire abroad. Those are one of the driving factors. (p29, Male)’* *So, if we have more collaboration and especially with those outside Nigeria … .even if they can’t come physically, they can contribute to our professional development. (p41, Male)*
No intention to migrate (motivation for staying back in Nigeria)	Sense of duty and patriotism	‘*I don’t think that everybody is meant to migrate. I mean, some people have to stay back as the last patriots, you know, until it gets completely unbearable. (p49, Female)’* *“Whether Nigeria is encouraging, whether it’s interesting to stay or not, I don’t think that will push me to just migrate and leave. This is our country and somehow, we have to make it work.(p39, Male)*
	Family ties and responsibilities	*‘We are a family of nine … I thought within myself that it is important that one of us that can take key decisions stayed back. Both my parents are still alive … I thought that we needed somebody very powerful amongst us to stay back (p26, Male)’*. *‘I’m not willing to go unless my family goes … my husband presently is not a fan of going abroad … if he’s here, my children are here, what am I going to do over there? (p48, Female)’*
	Thriving businesses	*‘the urge to leave is not as much as compared to somebody who is just a salary earner … because my business, all my future investments are here, so I cannot just leave (p32, Male)’*. *I started a business a few years back and it’s still thriving. And since I already have a business that is growing, I feel like it will give me more fulfilment (p49, Female)”*
	Professional and age considerations	*‘I’m just about 4 years away from becoming a professor. So, I can’t leave at the middle of it … I’m in my early forties already, so going back to school, trying to find my bearing again, to enter the system abroad would take like another 3 to 4 years. If I stay back, by then I would have become a [full] professor, and as a professor I can go for Sabbatical anyway (p39, Male)’*

#### Perspectives of those who had already migrated (potential enablers of return migration)

Overall, most participants who had already migrated expressed a willingness to return to Nigeria; however, this was generally framed as a long-term plan, often considered only after retirement. Based on the KIIs, five major themes emerged as potential enablers of return migration: improvement in security, economic improvement and better remuneration, healthcare system development, social infrastructural development, and community and family ties (Table 3).

Security concerns were a dominant theme, with many participants expressing a deep-seated fear of returning to Nigeria due to widespread insecurity. Some shared personal experiences or those of colleagues, emphasizing that without significant improvements in security, returning would not be a viable option. Economic challenges, particularly the high cost of living and low salaries, were also frequently cited. Participants highlighted the need for a more stable economic environment and competitive remuneration as crucial factors that could make return migration more attractive.

The state of the healthcare system was another major concern. Participants consistently described it as underfunded and often lacking essential infrastructure, which negatively impacts both patient care and working conditions for healthcare professionals. Many emphasized that significant investment in healthcare infrastructure and professional working conditions would be necessary to encourage their return. Beyond professional considerations, participants also underscored the importance of social infrastructure development, including improved education, transportation, and housing systems that could support their families’ well-being. They noted that returning to Nigeria would only be feasible if the quality of life could align, to some extent, with what they had become accustomed to abroad.

#### Perspectives of those intending to migrate (potential enablers of reconsidering migration intentions)

Four major themes emerged as potential enablers of reconsidering migration among those with intention migrate: improvement in security; economic improvement and better remuneration; enhanced healthcare infrastructure; and improved training and professional development (Table 3). Similar to those who have already migrated, improvement in security was repeatedly described as a significant factor that could make them reconsider their migration intention.

Improved overall economic conditions, including better salaries, were deemed crucial for reconsidering their decisions to leave Nigeria. Many respondents articulated dissatisfaction with their current benefit packages, compounded by inflation. The need for better healthcare facilities and resources was a recurring theme in the interviews. There was a clear indication that the current state of health facilities is inadequate with grueling work schedules, resulting in overworked health professionals. The need for improved training and professional development was one of the key issues that must be addressed to curtail migration intentions. This was predominantly discussed in terms of a poor training environment and the absence of international collaborations.

#### Perspectives of those with no intention to migrate (motivation for staying back in Nigeria)

Four major themes emerged as underlying motivations for staying back in Nigeria: a sense of duty and patriotism, family ties and responsibilities, thriving businesses, and professional and age considerations (Table 3). Many participants expressed a strong sense of duty and loyalty to Nigeria, emphasizing their commitment to contribute to nation-building rather than seeking opportunities abroad. Concerns about family obligations and emotional ties to loved ones were frequently cited as key motivation for staying back in Nigeria. The ownership of a thriving business was another key underlying motivation for staying back in Nigeria. Additionally, participants expressed concerns about the professional challenges they might face if they migrated abroad, fearing they may struggle to integrate into a new system. This concern is closely linked to considerations of advancing age, which also motivated most participants to stay back in Nigeria.

### Convergence between quantitative and qualitative findings across groups

**Figure 2.**
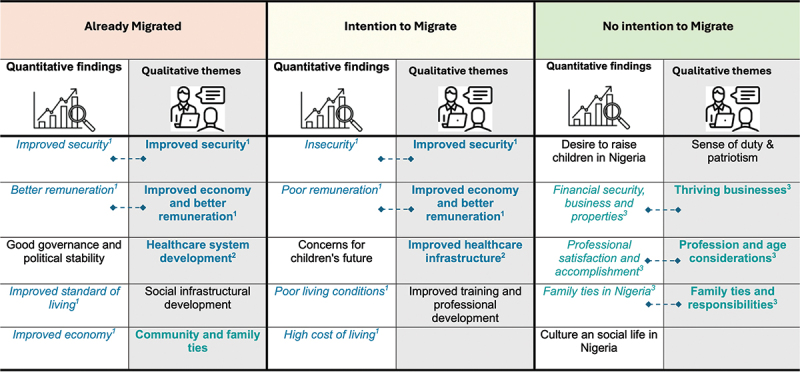
Areas of convergence between quantitative findings and qualitative themes on potential enablers of return migration and non-migration based on key informant interview of the three groups.

Overall, there was notable convergence between the qualitative and quantitative findings and across the different migration groups. Three qualitative enablers of potential return migration and reconsideration of migration intentions identified through the KIIs (Table 3) were consistent with the quantitative determinants of return migration and intention to migrate observed in the broader study population (Figure 1(a,b,d)). These overlapping factors included improved security, economic advancement through better remuneration, and healthcare system development (Figure 2). Likewise, three of the four major themes emerging from the KIIs exploring motivations for staying back in Nigeria (Table 3) aligned with the quantitative findings on drivers of non-migration (Figure 1(c)). These shared factors included thriving private businesses, professional and age-related considerations, and strong family ties (Figure 2).

Only the top five most prevalent quantitative findings were included in the figure. The quantitative findings are not ordered based on their prevalence, rather, they were arranged to align with the proposed solutions.

## Discussion

The migration of skilled healthcare workers continues to strain already fragile health systems, weakens service delivery, and hinders progress toward Universal Health Coverage (UHC). In this context, it is important to understand the drivers of migration as well as the conditions that support workforce non-migration or could incentivize return migration. To our knowledge, our study is the first empirical mixed-methods study in sub-Saharan Africa that explicitly explores the enablers of non-migration and return migration among medical professionals from a well-characterized graduating cohort, offering valuable insight for evidence-informed workforce planning and policy. Across the three groups studied, there was substantial convergence in perspective, particularly among those intending to migrate and those who had already migrated, around macro-level enablers, such as improved security, better remuneration, and stronger health system infrastructure, while those choosing to stay back in Nigeria placed greater emphasis on micro-level factors including family ties, personal fulfilment, and professional stability as their motivation. In this regard, those who had migrated or intended to do so appeared to prioritize broader systemic and policy-level improvements, whereas those who chose to stay seemed to anchor their decisions in personal or contextual factors, reflecting the complexity and diversity of decision-making across the three groups.

We found that half of this cohort had already migrated within 15 years of qualifying, and six in 10 of those still residing in Nigeria were actively seeking opportunities to leave. Although reliable national data on migration rates remain scarce [25], this finding is consistent with a previous study of 913 Nigerian physicians, where 80% of respondents reported intentions to migrate and were actively exploring options [26]. Nigeria, Africa’s most populous country, is facing a growing crisis in its health workforce. It also has one of the highest maternal mortality ratios in the world, with 1047 deaths per 100,000 live births, ranking just behind South Sudan and Chad [27]. The critical shortage of healthcare workers, driven in part by high rates of outmigration, contributes to persistently poor health outcomes. Addressing this challenge requires urgent and sustained efforts to retain skilled professionals within Nigeria and across the African continent. Beyond the direct health implications, the economic impact is also profound. Modelling studies project that, if the current migration trends continue, Africa could face cumulative economic losses of approximately 1.4 trillion US dollars by 2063 due to insufficient health workforce densities [5]. Even in a more optimistic scenario with improvements in non-migration, losses are still projected to reach 431 billion US dollars. These estimates make a strong case for urgent and sustained investment in strategies to reverse the current migration trajectory and secure both health and economic gains.

Building on the urgency to retain health workers, it is equally important to understand the dynamics of return migration. While only a small proportion of healthcare workers return to their countries of origin, previous studies have shown that many express a desire to do so [28]. In our study, majority of those who had already migrated expressed a willingness to return to Nigeria. However, this was often framed as a distant, long-term plan, typically linked to retirement. Although this suggests some openness to return, it raises concern that these highly skilled professionals may spend their most productive years serving in foreign health systems, returning only after they are no longer active in clinical practice. As a result, they may not contribute meaningfully to addressing Nigeria’s current health workforce shortages. This finding aligns with earlier research showing that many migrant health workers from sub-Saharan Africa working in Europe considered return migration only after their professional careers had ended, viewing it as a post-retirement option [29]. The observed pattern underscore the need to better understand the specific enablers of return migration in order to inform effective strategies that can incentivize earlier return and reintegration.

Literature on the enablers of non-migration and return migration remains limited in Nigeria. However, studies from other countries have reported that macro-level factors such as improved security, economic stability with better remuneration and functional healthcare system are the common enablers of non-migration and return migration [28,29]. From the qualitative data, participants who had already migrated and those intending to migrate highlighted these same macro-level enablers as conditions that could motivate their potential return migration and support remaining in Nigeria. These qualitative findings were consistent with the top five drivers of return migration and migration intention identified in our quantitative analysis. This convergence across both strands of data and among two different groups underscores the strength of applying a mixed-methods approach to address a complex and multifaceted issue [30]. The fact that all the identified enablers were macro-level factors indicates that meaningful action requires a coordinated, largely government-led response.

In 2024, Nigeria made a significant step by introducing a stand-alone National Policy on Health Workforce Migration [25]. The policy reflects a proactive approach to tackling the root causes and impacts of health worker emigration. Among its strategic components is a proposed collaboration between the Nigerians in Diaspora Commission and the Ministry of Interior to track emigration and return migration [25]. While the policy rightly acknowledges improved remuneration as a strategy to support both non-migration and return migration, it offers limited attention to broader issues such as improvement in national security and economic stability, factors that were consistently emphasized as enablers of return migration and non-migration in this study [31]. These limitations are understandable, given that these complex issues extend beyond the direct purview of the Ministry of Health and Social Welfare. Nonetheless, the policy’s limited empirical grounding in the lived experiences of healthcare professionals is a gap. In this respect, our study provides much-needed evidence to inform and refine strategies for facilitating return migration and non-migration of health workers within the Nigerian health system.

While it might be too early to determine the impact of Nigeria’s new National Policy on Health Workforce Migration, lessons can be drawn from other African countries that have made tangible progress in reversing the health worker ‘brain drain.’ For over a decade, Zambia has implemented sustained efforts to improve conditions of service for health workers [5]. These efforts included significant salary increases and improvements in working conditions to create a more supportive and enabling professional environment as indicated in its National Human Resources For Health Strategic Plan [32]. Zambia’s experience illustrates the effectiveness of investing in both financial and non-financial incentives to enhance health workforce non-migration [5]. It demonstrates that reversing brain drain is possible when health worker well-being and professional satisfaction are prioritized. Similarly, Ethiopia has achieved notable success in retaining its health workforce by offering comparatively better working conditions than many of its neighboring countries, fostering a merit-based professional culture, and providing fair compensation [5]. Between 2024 and 2028, Ethiopia has set an ambitious goal to quadruple its health workforce, underscoring a strong commitment to achieving UHC [5]. These strategies have unfolded alongside steady economic growth, further reinforcing the importance of economic stability as a key enabler of health worker return migration and non-migration [33], consistent with the findings from our study. As Nigeria begins to implement its new policy, these examples provide valuable insights to guide further actions.

The fact that the underlying motivations for the group that remained in Nigeria with no intention to migrate were primarily micro-level factors further shows that non-migration within this cohort was driven almost entirely by personal convictions rather than by effective government-led policies. While individual values, such as a sense of duty, patriotism, and family responsibilities, are commendable and important for nation-building, they are exhaustible. Research has shown that when structural conditions remain poor, such intrinsic motivations may eventually erode, especially under persistent poor working environments, insecurity, and economic hardship [33–35]. In the absence of meaningful systemic improvements, those who have chosen to stay may become disillusioned and join the migration train, thereby reinforcing the cycle of workforce depletion. Therefore, addressing the macro-level drivers of emigration, such as working conditions, remuneration, and national security must become an urgent policy priority. Sustainable retention of health workers cannot rely solely on personal sacrifice; it must be supported by structural reforms and tangible investments in the health workforce.

This study has several important strengths. To our knowledge, it’s the first sequential explanatory mixed-methods study of a medical and dental cohort in sub-Saharan Africa to investigate actual migration outcomes, intentions, and return migration among medical graduates 15 years post-graduation. This offers a rare longitudinal perspective on physician mobility in a high-emigration setting. Another key strength is the sequential explanatory mixed-methods design. This allowed for a comprehensive analysis, integrating quantitative data with in-depth qualitative insights to explain the underlying motivations behind migration decisions. Furthermore, the quantitative component achieved a high response rate. The qualitative component also purposefully included diverse perspectives across migration statuses, sex, geographic location, and training background, thereby enhancing the credibility and transferability of our findings.

However, the study also has limitations. First, our findings may not be generalizable to all Nigerian-trained doctors or other medical graduates across Africa, particularly given regional variations in exposure and macro-socioeconomic factors. Second, relying on self-reported quantitative data might have introduced recall or social desirability biases, especially concerning sensitive issues like migration intentions or dissatisfaction with working conditions. Third, although qualitative data saturation was achieved, interviews were conducted virtually. This means we might have missed important non-verbal cues that are typically visible in face-to-face interactions. Despite these limitations, this study offers timely and policy-relevant evidence that can inform national and regional strategies to support the return migration and improve non-migration of skilled health professionals.

## Supplementary Material

STROBE checklist.docx

Supplementary_appendix_clean.docx

## Data Availability

The quantitative and qualitative dataset generated and analyzed during this study is not publicly available due to the need to protect participant confidentiality. However, anonymized versions can be made available upon reasonable request from the corresponding author.
