# Correction

**DOI:** 10.1080/16549716.2026.2650063

**Published:** 2026-03-31

**Article title:** ‘This is our country and somehow, we have to make it work’: a sequential explanatory mixed-methods study of the enablers of non-migration and return migration in a cohort of Nigerian medical doctors and dentists

**Authors:** Author, A. N., Author, A. N., & Author, A. N.

**Journal:** *Global Health Action*

**Bibliometrics:** Volume 19, Number 1, e2623345

**DOI:** http://dx.doi.org/10.1080/16549716.2026.2623345

When this article was first published, the Paper Context section included information relating to another article.

This has now been corrected.

