# Supplementary material for: `This is our country and somehow, we have to make it work’: a sequential explanatory mixed-methods study of the enablers of non-migration and return migration in a cohort of Nigerian medical doctors and dentists
Source: Glob Health Action. 2026 Mar 20;19(1):2623345. doi: 10.1080/16549716.2026.2623345 (PMC13007456; doi:10.1080/16549716.2026.2623345)
Supplement: Supplementary_appendix_clean.docx [file ZGHA_A_2623345_SM9852.docx]

**Supplementary material**

**“This is our country and somehow, we have to make it work”: a sequential explanatory mixed-methods study of the enablers of retention and return migration in a cohort of Nigerian medical doctors and dentists**

# Quantitative study questionnaire

Study Number: |_|_|_|_|_|_|_|_|_|_|

Section 1- Demographic Information

- 1. Age (in years) as at the last birthday?

Ans: drop down with numbers 35 to 65.

- 1. What is your gender?

(Multiple choice, one response allowed)

- Male
- Female
  1. What is your marital status?

(Multiple choice, one response allowed)

- Single
- Married
- Separated
- Divorced
- Widowed
  1. What year did you get married

Ans: drop-down choice with years

- 1. What is the size of your nuclear family?

Ans: drop-down choice with numbers 1 to 15.

- 1. How many other dependents do you have, not counting spouses or children?

Ans: drop-down choice with numbers 0 to 15.

Section 2- Career Paths

- 1. What degree was awarded to you upon graduation from the University of Benin in 2008?

(Multiple choice, one response allowed)

- Bachelor of Dental Surgery (BDS)
- Bachelor of Medicine, Bachelor of Surgery (MBBS)
  1. Did you commence any postgraduate training?

(Multiple choice, one response allowed)

- Yes

- No (If no, skip to question 2.4)

- 1. What type of postgraduate training did you undertake? Tick all that apply.

(Multiple choice, multiple responses allowed)

- Residency/Fellowship (Will answer questions 2.3.1.1 to 2.3.1.)

- Masters

- PhD

- Other (Free text option)

- - 1. If you ticked Residency/Fellowship, please answer the following questions.
       1. What residency/fellowship training did you undertake?

Ans: Free Text

- - - 1. How many years post-MBBS/BDS did training commence?

Ans: drop-down choice with numbers 0 to 15.

- - - 1. Regarding residency training- Please pick the option that applies to you, multiple choice with branching questions)

- - 1. If you ticked Masters, please answer the following questions.
       1. What master’s degree(s) did you undertake? Option to add more than one response.

Ans: free text

+ Add another.

- - - 1. How many years post-MBBS/BDS did training commence?

Ans: drop-down choice with numbers 0 to 15.

- - - 1. For master’s degree training- Please pick the option that applies to you, multiple choice with branching questions)
    1. If you ticked Ph.D., please answer the following questions.
       1. What Ph.D. degree did you undertake? Option to add more than one response.

Ans: free text

+ Add another.

- - - 1. How many years post-MBBS/BDS did training commence?

Ans: drop-down choice with numbers 0 to 15.

- - - 1. For PhD degree training- Please pick the option that applies to you, multiple choice with branching questions)
    1. If you ticked other training, please answer the following questions.

2.3.5.1 What other postgraduate degree did you undertake?

Ans: free text

+ Add another.

- - - 1. How many years post-MBBS/BDS did training commence?

Ans: drop-down choice with numbers 0 to 15.

- - - 1. For other postgraduate degree training- Please pick the option that applies to you, multiple choice with branching questions)
  1. Are you in clinical practice, i.e., do you see actively patients?

- Yes

- No (If no, skip to question 2.5)

- - 1. If you answered yes to Question 2.4, on average, what percentage of your job is clinical practice? (Multiple choice, single response allowed)
- Less than 25%
- About 50%
- About 75%
- Practically 100%
  1. What is your current **PRIMARY** role?

Ans: free text option

- 1. How many years have you been in your current role?

Ans: drop-down choice with numbers 0 to 15.

- 1. Do you have other secondary roles? If so, please indicate (allow multiple responses)

- I do not have any other roles

- Teaching

- Research

- Administrative

- Other (free text option)

- 1. What **best** describes your **PRIMARY** job sector? (Multiple choice, single response allowed)
- Government
- Research or teaching institutions
- Private Office
- Non-Governmental Organizations (NGO)

Section 3- Current location

- 1. Do you currently live in Nigeria?

(Multiple choice, one response allowed)

- Yes (If yes, go to question 3.3)

- No

- 1. What country do you live in? (Single response allowed)

Ans: drop-down choice with list of countries

- - 1. What year did you leave Nigeria?

Ans: drop-down option with choice of years 2008 to 2023 (Skip to Section 4)

- 1. Do you intend to leave Nigeria in the future?

- Yes

- No (If no, skip to section 5)

- - 1. What is your likely destination country? Pick up to 3 if applicable.

Ans: drop-down choice with list of countries

Section 4: Drivers of out-migration/intention-to-migrate.

4.1. What factors in Nigeria influenced your decision to leave Nigeria or intention to leave to work in another country? (Pick the factors that apply to you)

- Unemployment/underemployment

- Insecurity

- Political situations in the country

- poor living conditions

- High cost of living

- Lack of job satisfaction

- Poor career development opportunities/prospects

- Poor quality of training

- Corruption in the health sector including nepotism

- Poor remuneration

- Hostile working environment

- Excessive workload

- Lack of professional support from supervisors/colleagues

- Poor facilities

- lack of optimal technology

- Peer pressure

- Family pressure

- personal ambition/desire to settle abroad

- concerns for children's future

**Others:** please list (free text)

4.2 What factors influenced the choice of the destination country to which you migrated or intend to migrate to?  (Pick the factors that apply to you)

- Security/Stability

-Conducive immigration and settlement policies in the host country including offer of permanent residency or citizenship

- Active recruitment of health workers’ relocation assistance

- Better remuneration

- Scholarships

- Better working environment

- Professional advancement opportunities

- Better quality of training/Improved knowledge and skills

- research opportunities

- Easy of finding a job abroad

- Better job satisfaction

- Professional status/prestige

- Job security

- No qualifying exams are required to practice

- Better quality of life including psychological wellbeing

- Peer Influence

- Adventure/ Taste of something new

- Family members abroad

- weather

- Previous travel abroad for professional reasons

- Social and cultural factors including language, cultural similarities, or personal freedom

**Others:** please list (free text)

4.3 Did you consider other countries for migration?

(Multiple choice, one response allowed)

- Yes

- No (If no, go to section 6)

4.3.1 Which other countries did you consider for migration? Pick up to 3 if applicable.

Ans: drop-down choice with list of countries

4.3.2 What factors discourage(d) you from migrating to those countries? (Pick the factors that apply to you)

- racism

- language barrier

- religion and culture

- qualifying examinations

- poor career prospects including a lack of information about career pathway

- poor working conditions including long working hours

- Inadequate remuneration

- lack of professionalism and respect

- cost of migration/ financial barriers

- weather conditions

- Poor quality of life including fractured family life

- Lack of opportunities for family members including lack of job opportunities for spouse or educational opportunities for children

**Others:** please list (free text)

4.4 What factors will influence your decision to return to Nigeria?

Ans [Free text]

Section 5: Drivers of non-migration

What factors influenced your decision not to migrate out of Nigeria? (Survey end)

- Bonds
- Racism in other countries
- language barrier in other countries
- religion and culture of other countries
- Good remuneration in Nigeria
- Professional satisfaction including professional accomplishment in Nigeria.
- Opportunity for career advancement in Nigeria
- Job security in Nigeria
- Good working conditions in Nigeria
- qualifying examinations to practice in other countries
- poor career prospects including a lack of information about career pathway in other countries
- Poor working conditions including long working hours in other countries
- Inadequate remuneration in other countries
- Family ties in Nigeria
- Advancing age
- Culture and social life in Nigeria
- Desire to raise children in Nigeria.
- Religion in Nigeria
- Lack of necessary resources to migrate out of Nigeria.
- Having financial security including ownership of business or properties in Nigeria
- Patriotism including the desire to settle in Nigeria/optimism about the future in Nigeria.
- weather conditions in other countries
- Poor quality of life including fractured family life in other countries
- Lack of opportunities for family members in other countries including lack of job opportunities for spouses or educational opportunities for children

**Others: please list (free text)**

Section 6: Drivers of return migration

- 1. Did you ever migrate from Nigeria and **then RETURN**?

- Yes

- No (If no, skip to end of survey)

- 1. What year did you leave? (Single response allowed)
- drop-down option with choice of N/A, and years 2008 to 2023
  1. Which country did you go to?

Ans: drop-down choice with list of countries

- 1. What year did you return? (Single response allowed)

drop-down option with choice of N/A, and years 2008 to 2023

- 1. If you migrated out of Nigeria **AND RETURNED**, what factors influenced your decision to leave your destination country and return to Nigeria?

Ans [Free text]

Section 7: End of survey for all respondents

7.1 Would you like to be contacted for a key informant interview on this topic?

- Yes

- No

# Qualitative interview question guide

## **Research Question 1**

**Could you share your thoughts about the prospect of returning to Nigeria?**

## **Participants:**

Doctors who have migrated and are already in destination country

## **Question Guide**

### Initial Considerations:

1. **Have you at some point considered a return to Nigeria in the future?**

- What prompted these considerations?
- How frequently do these thoughts occur?

### Challenges and Limitations:

1. **What specific challenges or obstacles do you foresee in returning to Nigeria?**

- Can you elaborate on these issues
- How do these issues impact your decision-making process?

### Facilitating Factors:

1. **With the current situation in Nigeria, what specific improvements or changes would make you consider moving back?**

- Please elaborate on these areas?
- Could you discuss any personal or professional aspirations that might play a role?

### Future Outlook

1. **Do you see any potential changes in the Nigerian healthcare system that would make it more attractive for returnees?**

- What role do you think government or healthcare institutions could play in encouraging doctors to return?

### Advice to Colleagues

1. **Based on your experiences, what advice would you give to other doctors who are contemplating whether to stay in Nigeria or migrate?**

- What factors should they consider?
- What lessons have you learned from your own experience?

## **Research Question 2**

## ***What issues must be fixed to change the minds of doctors intending to migrate, and what factors made them stay back in Nigeria for longer***

## Participants

Doctors who have not migrated but have intention to migrate

## **Question Guide**

### Motivation to Migrate

1. **What are the primary reasons motivating your decision to consider migrating from Nigeria at this specific time?**

- Are there specific professional, economic, or personal reasons influencing your decision?
- How have these issues affected your work and life in Nigeria?

### Reasons for Staying this long

1. **Are there factors that have kept you in Nigeria this long?**

- Are there aspects of your job, national, or family life that have kept you in Nigeria?
- How do these factors balance against the reasons for wanting to migrate?

### Conditions for Change in Mind

1. **What issues do you believe need to be addressed that would make you reconsider your plans of leaving?**

- Can you suggest any specific actions or policies that could address these issues?
- How quickly do you think these changes need to occur to influence your decision?

### Advice to Colleagues

1. **Based on your experiences, what advice would you give to other doctors who are contemplating whether to stay in Nigeria or migrate?**

- What factors should they consider in making their decision?
- What lessons have you learned from your own experience of staying longer and your own contemplation of migration?

## **Research Question 3**

***What contextual issues made them stay back, or is making them not considering migration from Nigeria?***

## Participants

Doctors who have not migrated and do not have any intention to migrate

## **Question Guide**

### Factors Influencing the Decision to Stay

1. **Could you explain the factors that influenced your choice to stay in Nigeria?**

- Are there specific professional, personal, or societal reasons behind your decision?
- How do these factors impact your daily life and career satisfaction?

### Impact of Current Realities

1. **How do the current realities in Nigeria impact your perspective on staying in the country?**

### Potential for Change of Mind

1. **Can you describe any circumstances or changes that might make you reconsider your decision to stay in Nigeria?**

- What specific events or developments could influence you to consider migration?
- How likely do you think these changes are to occur in the near future?

### Retention Strategies

1. **In your opinion, what specific actions can be taken to help retain healthcare professionals in Nigeria?**

- What role should the government, healthcare institutions, and professional organizations play?

### Advice to Colleagues

1. **What advice would you give to other doctors who are contemplating whether to stay in Nigeria or migrate?**

- What key factors should they consider in their decision-making process?
- What insights have you gained from your own experience that might help them?

| **Supplementary Table 1:** Characteristics of Medical and Dental Graduates from the University of Benin Medical School 15 Years Post-Graduation (i.e., 2008 – 2024) by terminal degree (i.e., MBBS and BDS) and migration status | | | | | | | | | | | | |
| --- | --- | --- | --- | --- | --- | --- | --- | --- | --- | --- | --- | --- |
| **Variable** | **Medicine (MBBS)**  **(n = 225)** | | | | | | **Dentistry (BDS)**  **(n = 49)** | | | | | |
|  | **Not Migrated*** | | | **Total**  **Not Migrated**  (n=113) | **Total**  **Migrated**  (n=112) | p-value | **Not Migrated*** | | |  |  |  |
|  | **Intention to migrate** | | p-value |  |  |  | **Intention to migrate *** | | p-value | **Total**  **Not Migrated**  (n=26) | **Total Migrated**  (n=23) | p-value |
|  | **No**  n=44 | **Yes**  N=69 |  |  |  |  | **No**  (n=13) | **Yes**  (n=13) |  |  |  |  |
| **Age** |  |  |  |  |  |  |  |  |  |  |  |  |
| Median age (years) | 43 | 42 |  | 43 | 41 |  | 41 | 42 |  | 42 | 41 |  |
| Mean age (years) | 43.7 | 42.9 | 0.079 | 43.1 | 41.9 | 0.001 | 42.5 | 42.3 | 0.7913 | 42.3 | 41.1 | 0.038 |
| Age range (years) | 40-54 | 39-51 |  | 39-54 | 38-60 |  | 40-47 | 40-46 |  | 40-47 | 36-46 |  |
|  |  |  |  |  |  |  |  |  |  |  |  |  |
| **Sex, n (%)** |  |  | 0.842 |  |  | 0.105 |  |  | 0.420 |  |  | 0.990 |
| Female | 10 (29.4) | 19 (27.5) |  | 34 (30.4) | 46 (40.7) |  | 6 (46.2) | 4 (30.8) |  | 11 (40.7) | 9 (40.9) |  |
| Male | 24 (70.6) | 50 (72.5) |  | 78 (69.6) | 67 (59.3) |  | 7 (53.7) | 9 (69.2) |  | 16 (59.3) | 13 (59.1) |  |
|  |  |  |  |  |  |  |  |  |  |  |  |  |
| **Marital Status, n (%)** |  |  | 0.229 |  |  | 0.835 |  |  |  |  |  |  |
| Married | 29 (85.3) | 64 (92.8) |  | 101 (91.0) | 101 (90.2) |  | 11 (84.6) | 12 (92.3) | 0.539 | 24 (88.9) | 20 (95.2) | 0.430 |
| Not Married** | 5 (14.7) | 5 (7.2) |  | 10 (9.0) | 11 (9.9) |  | 2 (15.4) | 1 (7.7) |  | 3 (11.1) | 1 (4.8) |  |
|  |  |  |  |  |  |  |  |  |  |  |  |  |
| **Timing of Marriage** |  |  |  |  |  |  |  |  |  |  |  |  |
| Median year married | 2012 | 2012 |  | 2012 | 2012 |  | 2011 | 2012.5 |  | 2012 | 2011.5 |  |
| Range (year married) | 1993-2021 | 1998-2020 |  | 1993-2021 | 1996-2021 |  | 2004-2019 | 2007-2021 |  | 2004-2021 | 2009-2019 |  |
|  |  |  |  |  |  |  |  |  |  |  |  |  |
| **Dependents** |  |  |  |  |  |  |  |  |  |  |  |  |
| Nuclear family dependents (median) | 5 | 5 |  | 5 | 5 |  | 4 | 4.5 |  | 4 | 5 |  |
| Nuclear family dependents (range) | 0-10 | 1-8 |  | 1-10 | 1-9 |  | 1-8 | 2-7 |  | 1-8 | 0-6 |  |
| Non-nuclear family dependents (median) | 4 | 3 |  | 3 | 2 |  | 2 | 1 |  | 2 | 3 |  |
| Non-nuclear family dependents (range) | 0-16 | 0-15 |  | 0-16 | 0-21 |  | 0-9 | 0-6 |  | 0-9 | 0-10 |  |
|  |  |  |  |  |  |  |  |  |  |  |  |  |
| **Commenced Postgraduate Training?**  **n (%)** |  |  | 0.433 |  |  | 0.689 |  |  | 0.185 |  |  | 0.518 |
| Yes | 27 (79.4) | 59 (85.5) |  | 94 (83.9) | 97 (85.8) |  | 5 (38.5) | 2 (15.4) |  | 20 (74.1) | 4 (18.2) |  |
| No | 7 (20.6) | 10 (14.5) |  | 18 (16.1) | 16 (14.2) |  | 8 (61.5) | 11 (84.6) |  | 7 (25.9) | 18 (81.8) |  |
|  |  |  |  |  |  |  |  |  |  |  |  |  |
| **T*y*pe of Postgraduate training:** |  |  |  |  |  |  |  |  |  |  |  |  |
| ***Residency***  ***/Fellowship*** | 21 (77.8) | 51 (86.4) | 0.313 | 80 (85.1) | 91 (93.8) | 0.049 | 4 (50.0) | 10 (90.9) | 0.046 | 15 (75.0) | 10 (55.6) | 0.207 |
| Status of training: |  |  |  |  |  |  |  |  |  |  |  |  |
| Completed | 15 (71.4) | 25 (49.0) | 0.082 | 44 (55.0) | 58 (63.7) | 0.245 | 4 (100.0) | 5 (50.0) | 0.078 | 9 (60.0) | 5 (50.0) | 0.622 |
| Currently Enrolled | 6 (28.6) | 23 (45.1) | 0.194 | 32 (40.0) | 13 (14.3) | 0.001 | 0 (0) | 5 (50.0) | 0.078 | 6 (40.0) | 0 (0) | 0.022 |
| Discontinued | 0 (0) | 3 (5.9) | 0.256 | 4 (5.0) | 20 (22.0) | 0.001 | 0 (0) | 0 (0) | - | 0 (0) | 5 (50.0) | 0.002 |
|  |  |  |  |  |  |  |  |  |  |  |  |  |
| **Master’s degree** | 8 (29.6) | 16 (27.1) | 0.810 | 29 (30.8) | 27 (27.8) | 0.647 | 5 (62.5) | 5 (45.4) | 0.463 | 10 (50.0) | 8 (44.4) | 0.732 |
| Status of training: |  |  |  |  |  |  |  |  |  |  |  |  |
| Completed | 7 (87.5) | 12 (75.0) | 0.477 | 22 (75.9) | 23 (85.2) | 0.380 | 1 (20.0) | 3 (60.0) | 0.197 | 4 (40.0) | 8(100.0) | 0.007 |
| Currently Enrolled | 1 (12.5) | 2 (12.5) | 1.000 | 4 (13.8) | 4 (14.8) | 0.913 | 4 (80.0) | 2 (40.0) | 0.197 | 6 (60.0) | 0 (0) | 0.007 |
| Discontinued | 0 (0) | 2 (12.5) | 0.296 | 3 (10.3) | 0 (0) | 0.086 | 0 (0) | 0 (0) | - | 0 (0) | 0 (0) | - |
|  |  |  |  |  |  |  |  |  |  |  |  |  |
| ***PhD*** | 3 (11.1) | 5 (8.5) | 0.696 | 9 (9.6) | 5 (5.2) | 0.241 | 0 (0) | 0 (0) | - | 0 (0) | 3 (16.7) | 0.057 |
| Status of training |  |  |  |  |  |  |  |  |  |  |  |  |
| Completed | 0 (0) | 2 (40.0) | 0.206 | 3 (33.3) | 3 (60.0) | 0.334 | 0 (0) | 0 (0) | - | 0 (0) | 0 (0) | - |
| Currently Enrolled | 3 (100.0) | 3 (60.0) | 0.206 | 6 (66.7) | 2 (40.0) | 0.334 | 0 (0) | 0 (0) | - | 0 (0) | 2 (66.7) | - |
| Discontinued | 0 (0) | 0 (0) | - | 0 (0) | 0 (0) | - | 0 (0) | 0 (0) | - | 0 (0) | 1 (33.3) | - |
|  |  |  |  |  |  |  |  |  |  |  |  |  |
| ***Other Postgraduate Training****** | 3 (11.1) | 3 (5.1) | 0.309 | 6 (6.4) | 5 (5.2) | 0.716 | 1 (12.5) | 0 (0) | 0.228 | 2 (10.0) | 4 (22.2) | 0.302 |
|  |  |  |  |  |  |  |  |  |  |  |  |  |
| **Clinical Practice? n (%)** |  |  | 0.353 |  |  | 0.060 |  |  | 0.539 |  |  | 0.016 |
| No | 5 (14.7) | 6 (8.7) |  | 11 (9.8) | 21 (18.6) |  | 1 (7.7) | 2 (15.4) |  | 3 (11.1) | 9 (40.9) |  |
| Yes | 29 (85.3) | 63 (91.3) |  | 101 (90.2) | 92 (81.4) |  | 12 (92.3) | 11 (84.6) |  | 24 (88.9) | 13 (59.1) |  |
|  |  |  |  |  |  |  |  |  |  |  |  |  |
| **Percent Clinical Practice:** |  |  |  |  |  |  |  |  |  |  |  |  |
| ≤25% Clinical Practice | 5 (14.7) | 6 (8.7) |  | 2 (2) | 1 (1.1) |  | 0 (0) | 1 (9.1) |  | 1 (4.2) | 1 (7.7) |  |
| 50% Clinical Practice | 2 (6.9) | 0 (0) |  | 7 (6.9) | 5 (5.4) |  | 0 (0) | 1 (9.1) |  | 4 (16.7) | 1 (7.7) |  |
| 75% Clinical Practice | 0 (0) | 7 (11.1) |  | 34 (33.7) | 9 (9.8) |  | 5 (41.7) | 4 (36.3) |  | 7 (29.2) | 3 (23.1) |  |
| 100% Clinical Practice | 9 (31.0) | 22 (34.9) |  | 58 (57.4) | 77 (83.7) |  | 7 (58.3) | 5 (45.5) |  | 12 (50.0) | 8 (61.5) |  |
|  |  |  |  |  |  |  |  |  |  |  |  |  |

**Not Married includes people who were single, separated, divorced, or widowed. ***Other postgraduate training includes diploma courses or specialized clinical courses.
